# Mitoapocynin Attenuates Organic Dust Exposure-Induced Neuroinflammation and Sensory-Motor Deficits in a Mouse Model

**DOI:** 10.3389/fncel.2022.817046

**Published:** 2022-04-13

**Authors:** Nyzil Massey, Denusha Shrestha, Sanjana Mahadev Bhat, Piyush Padhi, Chong Wang, Locke A. Karriker, Jodi D. Smith, Anumantha G. Kanthasamy, Chandrashekhar Charavaryamath

**Affiliations:** ^1^Biomedical Sciences, Iowa State University, Ames, IA, United States; ^2^Veterinary Diagnostic and Production Animal Medicine (VDPAM), Iowa State University, Ames, IA, United States; ^3^Statistics, Iowa State University, Ames, IA, United States; ^4^Veterinary Pathology, Iowa State University, Ames, IA, United States

**Keywords:** organic dust, inflammation, microglia, neurodegeneration, mitoapocynin

## Abstract

Increased incidences of neuro-inflammatory diseases in the mid-western United States of America (USA) have been linked to exposure to agriculture contaminants. Organic dust (OD) is a major contaminant in the animal production industry and is central to the respiratory symptoms in the exposed individuals. However, the exposure effects on the brain remain largely unknown. OD exposure is known to induce a pro-inflammatory phenotype in microglial cells. Further, blocking cytoplasmic NOX-2 using mitoapocynin (MA) partially curtail the OD exposure effects. Therefore, using a mouse model, we tested a hypothesis that inhaled OD induces neuroinflammation and sensory-motor deficits. Mice were administered with either saline, fluorescent lipopolysaccharides (LPSs), or OD extract intranasally daily for 5 days a week for 5 weeks. The saline or OD extract-exposed mice received either a vehicle or MA (3 mg/kg) orally for 3 days/week for 5 weeks. We quantified inflammatory changes in the upper respiratory tract and brain, assessed sensory-motor changes using rotarod, open-field, and olfactory test, and quantified neurochemicals in the brain. Inhaled fluorescent LPS (FL-LPS) was detected in the nasal turbinates and olfactory bulbs. OD extract exposure induced atrophy of the olfactory epithelium with reduction in the number of nerve bundles in the nasopharyngeal meatus, loss of cilia in the upper respiratory epithelium with an increase in the number of goblet cells, and increase in the thickness of the nasal epithelium. Interestingly, OD exposure increased the expression of HMGB1, 3- nitrotyrosine (NT), IBA1, glial fibrillary acidic protein (GFAP), hyperphosphorylated Tau (p-Tau), and terminal deoxynucleotidyl transferase deoxyuridine triphosphate (dUTP) nick end labeling (TUNEL)-positive cells in the brain. Further, OD exposure decreased time to fall (rotarod), total distance traveled (open-field test), and olfactory ability (novel scent test). Oral MA partially rescued olfactory epithelial changes and gross congestion of the brain tissue. MA treatment also decreased the expression of HMGB1, 3-NT, IBA1, GFAP, and p-Tau, and significantly reversed exposure induced sensory-motor deficits. Neurochemical analysis provided an early indication of depressive behavior. Collectively, our results demonstrate that inhalation exposure to OD can cause sustained neuroinflammation and behavior deficits through lung-brain axis and that MA treatment can dampen the OD-induced inflammatory response at the level of lung and brain.

## Introduction

Agriculture is one of the biggest drivers of the economy in the United States of America (USA) and worldwide. The agriculture sector employs around 1.3 billion people worldwide (International Labor Organization, 2018), underscoring the importance to the world economy. Growing demand for cheaper source of protein has transformed the animal production into large-scale industrial operations referred to as concentrated animal feeding operations (CAFOs) (reviewed in Sethi et al., 2017; Nordgren and Charavaryamath, 2018). People working in CAFOs are exposed to many onsite air contaminants and suffer from respiratory symptoms and decline in lung function (Sethi et al., 2017). Organic dust (OD) is one of the major airborne contaminants found in the agriculture industry. Extensive research efforts on the effects of OD exposure on the lungs is already underway (Charavaryamath and Singh, 2006; Bhat et al., 2019), but inadequate data is available for the OD exposure effects on the brain. OD is a mixture of gases (methane, ammonia, and hydrogen sulfide), particulate matter (0.01–1,000 μm), and microbial products such as lipopolysaccharide, peptidoglycan, fungal spores, and many other contaminants (Iowa State University and University of Iowa, 2002).

Exposure to previously known air contaminants, such as particulate matter, pesticides, and diesel exhaust, have been associated with a decrease in neurocognitive function, mainly affecting brain regions involved in memory and complex cognition (Liu et al., 2003; Chen et al., 2017; Ehsanifar et al., 2019). For example, a recent study has indicated a link between an exposure to above-normal levels of polluted air and an increased risk of dementia (Cacciottolo et al., 2017). Further, proteinopathies usually associated with Parkinson's disease (PD) and Alzheimer's disease [AD; hyperphosphorylated Tau (p-Tau) and amyloid-beta plaques] have been found in the brains of children and young adults exposed to the chronic air pollution (Calderón-Garcidueñas et al., 2012; Cacciottolo et al., 2017; Kilian and Kitazawa, 2018). Also, a variety of behavioral and cognitive changes indicative of neurodegenerative disorders (Trojsi et al., 2018) have been associated following chronic inhalation of air contaminants (Salvi and Salim, 2019).

Interestingly, despite a very low diesel exhaust levels found in rural communities and a regulated approach to safer pesticides in modern agricultural practices (United states Environmental protection Agency), the incidences of PD are 2–10 times greater in the agricultural belt of the American Midwest and northeast regions than the rest of the country (Wright Willis et al., 2010). It is possible that exposure to OD within and near to agriculture production facilities could be one of the etiologies. Recent data from U.S. Geological Survey (USGA) confirms the presence of contaminants in the farming (mid-western) area of the USA (U.S. Geological Survey, 2021). When compared to the other air contaminants, it is possible that OD can be far more potent in evoking similar inflammatory response and neurobehavioral changes in the brain due to the presence of multiple pathogen-associated molecular patterns (PAMPs) in addition to the particulate matter commonly present in other air pollutants. Mechanistically, air contaminants have the potential to reach the brain directly via olfactory nerve axons following inhalation. On the other hand, air contaminants have been shown to indirectly affect the brain through systemic route as well (Peters et al., 2006; Oberdörster et al., 2009).

Microglia are the endogenous resident immune cells of the brain and have been shown to acquire an inflammatory phenotype upon exposure to air contaminants (Levesque et al., 2011). Microglia, like other innate cells, recognize various danger signals through pattern recognition receptors (PRRs) (Saijo et al., 2013) and sensory channels (Sarkar et al., 2020). Activated microglia have been shown to release peroxynitrite through voltage gated potassium channels (KCNA3) which are neurotoxic in nature (Fordyce et al., 2005). In our previous novel approach, we demonstrated that OD exposure induces inflammatory phenotypes in microglia (*in vitro*) and resultant inflammation involved HMGB1 and RAGE signaling (Massey et al., 2019). HMGB1, a nucleosome protein, is a damage-associated molecular pattern (DAMP) that is ubiquitously present in all the nucleated cells. HMGB1 is known to undergo nucleocytoplasmic translocation and secretion into extracellular milieu under the influence of inflammagens. Secreted HMGB1 acts as a proinflammatory stimulus and exacerbates the inflammatory process. Out of 14 identified receptors of the HMGB1, receptor for advanced glycation end products (RAGE) appears to be the main receptor for HMGB1 (Ugrinova and Pasheva, 2017). Ethyl pyruvate (EP) inhibits the post-translational modification of HMGB1 and thus renders it unable to translocate from nucleus into the cytosol (Shin et al., 2015). EP prevented OD-exposure-induced nucleocytoplasmic translocation of HMGB1 in microglia and, overall, curtailed the inflammatory responses. Further, EP treatment significantly prevented the microglia from changing to an M1 phenotype from their resting M0 phenotype.

Mitochondria are emerging to be essential organelles for cellular respiration and their role in neuroinflammation due to exposure to toxicants (Prakash et al., 2017; Sarkar et al., 2017). Next, we demonstrated how OD exposure induces mitochondrial damage in exposed microglia and secretion of mitochondrial DNA (mtDNA). Subsequently, we demonstrated that cyclic GMP-AMP synthase-stimulator of interferon genes (cGAS-STING) pathway could be targeted to reduce mtDNA driven and OD-exposure-induced inflammation.

We employed *in vitro* and *ex vivo* models (Massey et al., 2021a) to understand how OD-exposure induces mitochondrial dysfunction. Mitoapocynin (MA) is a mitochondrion targeting cytoplasmic NOX-2 inhibitor with proven effectiveness in reducing toxicant exposure-induced mitochondrial damage (Langley et al., 2017). MA is a highly effective synthetic analog of apocynin that is synthesized by conjugating a triphenylphosphonium cation moiety via an alkyl chain with differing chain lengths (C2-C11). Neuroprotective effects of MA(C2) have been demonstrated using *in vitro* models (Ghosh et al., 2016), and the long-acting MA(C11) has been used in *in vivo* models (Dranka et al., 2014; Langley et al., 2017). MA treatment of exposed microglia significantly reduced mitochondrial stress responses and prevented secretion of mt-DNA into the cytosol, thereby reducing exposure induced inflammation and mitochondrial damage (Massey et al., 2021a).

Despite our recent published data on the OD-exposure effects on microglial cells, whether inhaled OD would induce inflammation in the brain remains largely unknown. To fully understand the pathophysiology of the inhaled OD exposure effects, the use of an *in vivo* model is required.

In the current manuscript, we tested a hypothesis in that inhaled OD would induce inflammation in the brain with sensory motor deficits and orally administered MA will be protective. To test this hypothesis, we used a mouse model of OD exposure and investigated neuroinflammatory and neurodegenerative potential. We explored how the inhaled dust would travel to the brain and other organs by using fluorescently labeled lipopolysaccharide (LPS) as a tool. Next, we examined the various areas of the brain that are affected due to OD exposure. To understand the impact of inflammation in the brain on sensory-motor abilities, we subjected mice to a battery of behavioral tests. Using cytoplasmic NOX-2 inhibitory agent MA(C11), we demonstrated a reduction in OD-induced neuroinflammation and neurodegeneration with improvement in neuro-motor and neuro-sensory deficits.

## Materials and Methods

### Preparation of Organic Dust Extract

All the experiments were conducted in accordance with an approved protocol from the Institutional Biosafety Committee (IBC, protocol# 19-004) of the Iowa State University. Settled swine barn dust (representing OD) samples were collected from various swine production facilities, placed in sealed bags with a desiccant, and transported on ice to the laboratory and prepared as previously described (Massey et al., 2021b). The prepared stock (100%) was diluted in cell culture medium to prepare a 1% (v/v) solution to use in our experiments (Supplementary Table 1). We have been quantifying the LPS content of our organic dust extract (ODE) samples using Pyrochrome® Kinetic Chromogenic Endotoxin (CAPE COD, catalog # CG-1500-5) as per the instructions. Previously, we have published the LPS content of several of our ODE samples (Bhat et al., 2019).

### Chemicals and Reagents

Dulbecco's minimum essential medium (DMEM), penicillin, streptomycin (PenStrep), L-glutamine, and trypsin-EDTA were purchased from Life Technologies (Carlsbad, CA). Fetal bovine serum (FBS) was purchased from Atlanta Biologicals (Flowery Branch, GA, catalog # S11150H and lot # A17002). DNA purification kit (Thermo Fisher Scientific, catalog # K0512) were purchased from Thermo Fisher Scientific (Waltham, MA). MA was procured from Dr. Balaraman Kalyanaraman (Medical College of Wisconsin, Milwaukee, WI) and was dissolved (3 mg/kg) in 10% ethanol in normal saline (Supplementary Table 1). Fluorescent LPS was purchased from Thermo Fisher Scientific (catalog # L23351).

### MA Dosage for Intranasal Exposure of Mouse

The dose rate of MA for our current work was carefully chosen based on the factors such as severity of the model and the therapeutic efficacy reported in other previous *in vivo* model systems. MA, when given orally for 3 mg/kg/day for shorter duration studies (1–6 days) (Ghosh et al., 2016) and 3 mg/kg for 3 days a week for longer duration (3–4 weeks) (Dranka et al., 2014), has been shown to rapidly cross the blood brain barrier and persist in the central nervous system (CNS) to prevent the hyposmia and loss of motor function in mice. Whereas, in more severe models, such as MitoPark mice, a higher dose of 10 mg/kg has been proven to be more effective. Since we employed wild type C57BL/6 mice for a 5-week ODE exposure model, we administered MA at 3 mg/kg for 3 days a week.

### Mouse Model of ODE Exposure

All the animal experiments were conducted as per approval of the Institutional Animal Care and Use Committee (IACUC) protocols at the Iowa State University. These protocols were designed in accordance with federal guidelines on the use of animals in the biomedical research. Eight-week-old male C57BL/6N mice (11–12 animals per group), obtained from Charles River, were housed under the following standard conditions: constant temperature (22 ± 1°C), humidity (relative, 30%), and a 12-h light/dark cycle. From the preliminary data, we estimated that a minimum of 10 animals per group was required to achieve a statistical power of 80% with α set at ≤ 0.05. All the mice were pre-screened for normal baseline performance during behavioral assessments conducted prior to randomly assigning animals to the experimental groups. The animals were randomly assigned to the treatment groups using the mean weight of the group as the criteria. Individual animals were assigned numbers and were distributed such that the mean weight of each group was not significantly different from the other groups. Investigators involved with collection of the data and analysis were blinded to group allocation of mice. After allowing an acclimatization period of seven days (0 week), mice were given either normal saline, fluorescent LPS, or 12.5% ODE intranasally (25 μl/nostril, 50 μl/mice) for 5 days a week (Monday-Friday) for a total of 5 weeks (Supplementary Table 1). Mice were also orally gavaged with 3 mg/kg of MA every other day (Monday/Wednesday/Friday) during the 5-week treatment regimen. Final behavioral performance was recorded on the last weekend of the 5-week treatment regimen, after which the mice were euthanized by administering CO_2_ using a pressurized chamber. Half of the animals in each treatment group were perfused with 4% paraformaldehyde, and perfused tissues were trimmed, processed, and embedded in paraffin. Upper respiratory tract tissues were decalcified and processed before embedding. Five-micron thick paraffin sections were prepared and mounted on glass slides and used in various staining and imaging.

### Quantification of Mucus Producing Goblet Cells

Mucus-producing goblet cells were quantified in the decalcified nasal epithelial sections stained with Hematoxylin and Eosin (H&E). Images were captured with the 20 × objective lens and were manually analyzed by an investigator blinded to the treatment groups. Mucus positive goblet cells appear as clear/empty when compared to other cells whose cytoplasm takes up the classical eosin stain. The goblet cells were identified, counted, and expressed as number of cells per mm of basement membrane.

### Quantification of Loss of Ciliated Epithelium

Ciliated epithelium was quantified in the nasal epithelial sections stained with H&E. Images were captured with the 20 × objective lens and were analyzed manually by an investigator blinded to treatment groups. Total length of ciliated nasal epithelium was measured in 5 randomly chosen fields per mouse (3 mouse/per treatment). Ciliated epithelium was plotted as a fraction of the total epithelium lining and all treatment groups were normalized to the control group.

### Western Blot

Sample preparation from fresh tissues, gel running, and transfer of the gel were performed as per established techniques (Massey et al., 2019). Briefly, fresh tissue samples were harvested from the animals after dissection, cut into small pieces with a scalpel, and placed in radioimmunoprecipitation assay (RIPA) buffer. Tissues were further triturated into a suspension and sonicated to obtain a whole cell lysate, and total protein content was quantified using Bradford assay. Equal amounts of proteins (20 μg/well) were resolved on 10% sodium dodecyl sulfate-polyacrylamide gel electrophoresis (SDS-PAGE) gels (Bio-Rad). Next, proteins were transferred to a nitrocellulose membrane, and the non-specific binding sites were blocked for an hour with a blocking buffer specially formulated for fluorescent western blotting (Rockland Immunochemicals, Pottstown, PA). Membranes were incubated overnight at 4°C with the respective primary antibodies (listed in Supplementary Table 2). Next, membranes were incubated with the respective secondary donkey anti-rabbit IgG highly cross-adsorbed (A10043) or anti-mouse 680 Alexa Fluor antibodies (A21058, Thermo Fisher Scientific). Membranes were washed three times with phosphate buffer solution (PBS) containing 0.05% Tween-20 and visualized on the Odyssey infrared imaging system. Using β-actin (1:5,000, Abcam; ab6276 or ab8227) as a loading control, band densities were normalized and densitometry was performed (ImageJ, NIH).

### TUNEL Assay

We performed terminal deoxynucleotidyl transferase deoxyuridine triphosphate (dUTP) nick end labeling (TUNEL) assay as per our published protocol (Massey et al., 2021b). Using formaldehyde perfused brain tissues, we prepared paraffin embedded tissue sections (10 μm). TUNEL assay was performed using a DeadEnd™ Fluorometric TUNEL System as per the manufacturer's instructions (Promega Corporation, Madison, WI, USA). Briefly, tissue sections were rinsed in PBS and incubated in 20 μg/ml proteinase K for 10 min. After rinsing in PBS (0.05 M phosphate buffer containing 0.145 M sodium chloride, pH 7.4), tissue sections were incubated with equilibration buffer and then with TdT enzyme in a humidified chamber at 37°C for 60 min. Next, tissue sections were transferred into pre-warmed working strength stop wash buffer for 15 min. Following rinsing with PBS, tissue sections were mounted with VECTASHIELD® antifade mounting medium containing 4', 6-Diamidino-2-Phenylindole, Dihydrochloride (DAPI, Vector Labs, Burlingame, California). Tissue sections on the slides were covered with a cover-glass. Nuclei were stained blue with DAPI, and localized green fluorescence of apoptotic cells was detected by fluorescence microscopy and photographed (Nikon Eclipse TE2000-U, Photometrics Cool Snap cf, HCImage). TUNEL positive cells were counted manually using ImageJ (NIH) software in five randomly selected microscopic fields viewed under 20X objective lens. Percent of TUNEL positive cells were quantified, statistically analyzed, and plotted.

### Immunohistochemistry (IHC)

Paraffin embedded tissue sections (10 μm) were deparaffinized with xylene and rehydrated using a decreasing gradient of ethanol. Heat-induced antigen retrieval was performed using citrate buffer (10 mM Citric acid, 0.05% Tween-20, pH 6.0) for 20 min at 90–95°C. To reduce non-specific binding, blocking was carried out with 10% Normal donkey serum (EMD Millipore, Burlington, Massachusetts) and 0.2% Triton-X100 in PBS for 1 h at room temperature. A list of all the primary and secondary antibodies used are listed in Supplementary Table 3. Primary antibodies were prepared in antibody diluent solution (2.5% normal donkey serum, 0.25% sodium azide, 0.2% trition X 100, and PBS, Abcam, Cambridge, Massachusetts), and sections were covered with primary antibodies overnight at 4°C. Next, each well was washed with PBS five times and then incubated with the respective secondary antibodies for 1 h, followed by mounting with VECTASHIELD® antifade mounting medium containing DAPI (Vector Labs, Burlingame, California) on glass sides. Slides were dried overnight and then imaged using a Nikon Eclipse C1 microscope.

### Quantification of IHC Staining

Five fields per slide were randomly chosen and total positively stained cells were counted (ImageJ). Then, total staining intensity per field (cy3 or Fluorescein isothiocyanate, FITC) was measured using a software (HC Image, Hamamatsu Corp, Sewickley, Pennsylvania). Total field's intensity (cy3 or FITC) was divided by the number of cells per field to obtain mean intensity (cy3 or FITC) per cell.

### Behavioral Measurements

An automated open field test for locomotor activity and the rotarod experiment for testing motor activity was performed as part of the behavioral measurements (Ghosh et al., 2010, 2013). Spontaneous movements of mice were automatically measured (AccuScan, model RXYZCM-16, Columbus, OH). A clear Plexiglas chamber (40 × 40 × 30.5 cm) with ventilation holes was used for measuring locomotor activity. Infrared sensors were placed every 2.54 cm along the perimeter with 16 infrared beams on each side and 2.5 cm above the floor. Two extra pair of 16 sensors were also placed 8 cm high from the floor on opposite sides. VersaMax Analyzer (AccuScan, model CDA-8, Columbus, OH) was used to collect the data and Versaplot software was used to analyze the data. Animals were acclimatized and trained for three consecutive days by placing them inside the chamber for 10 min daily before starting any treatments. In the open-field experiment, mice were monitored for horizontal activity, vertical activity, total distance traveled (cm), total movement time (s), and total rest time (s) over a 10-min test session. Before any treatments, mice were also trained for rotarod experiment for three consecutive days by letting them run on an accelerating profile for 3 min. Three trials for each mouse were conducted on an accelerating profile for 3 min. Each mouse was given a 5–7 min rest interval to eliminate stress and fatigue. Three rotations on the rotarod were considered as a fall. A baseline reading a day before starting of any treatment regimen was performed, and a final read data after 5 weeks was collected.

### Olfaction Test

Novel scent test (NST) was performed to evaluate the olfactory ability of the mouse. This test utilizes oblong plexiglass cage with video monitoring from above. NST quantifies the ability to discriminate between neutral water and an unfamiliar odor. Time spent sniffing, latency to enter sniffing region, and number of entries are determined using ANY-maze software and were defined as the animal's head coming within 1 cm of contact with the scented object. Trials each lasted for 3 min and the scents used included cotton-balls infused with oils of lemon, peppermint, or vanilla. Scented material was placed inside a cap from a 50 ml tube, and the cap was placed in a petri dish. In the NST, the scented side of the cotton swab was placed scent-side down. At the other end of the arena, cotton infused with plain, unscented water was placed and a mouse was placed in the middle of arena facing the water sample. Criteria, such as total distance traveled, total number of head entries to sniff zones, or total time spent immobile, were measured. A 3-day acclimatization followed by a baseline read was performed before any treatment.

### Quantification of Neurochemicals With HPLC

Brain tissue regions were micro-dissected and processed using an antioxidant buffer (30 μl/mg of tissue) containing 0.2 M perchloric acid, 0.5 mg/ml Na_2_S_2_O_5_,0.5 mg/ml EDTA, and Isoproterenol was used as an internal standard as previously described (Langley et al., 2017, 2018). The tissue samples were homogenized, sonicated for 2.5 min, and centrifuged for 10 min before filtering in a.2-micron nylon filter. Before high-performance liquid chromatography-electrochemical detection (HPLC-ECD) analysis, samples were diluted and loaded into auto-sampler vials.

### Chromatographic Conditions

The assay was validated in a previous method for HPLC-ECD detection of tyrosine and tryptophan-based metabolites (Langley et al., 2017). The brain tissue lysates were loaded in the auto-sampler (Thermo Scientific WPS-3000TSL). The analytes were separated isocratically with mobile phase (MDTM, Thermo-Scientific) by reversed-phase C-18 Column (ZORBAX Eclipse Plus 4.6 × 100 mm, Agilent) at 0.6 ml/min for 21 min (pump, ISO-3100 SD, Thermo Scientific). Detection of metabolites DA, NE, DOPAC, HVA, 5-HIAA, and 5-HT were performed by ECD (CoulArray 5600A) using the following potentials: Channel 1: 350 mV (ESA Guard cell, 5020) Channel 3: −150 mV and CH4: +220 mV (ESA Microdialysis cell, 5014B). Method development and Data integration and analysis were performed using Chromeleon (v. 7.1.3) and ESA CoulArray Software (v. 3.10).

### Statistical Analysis

**Figure 1:**
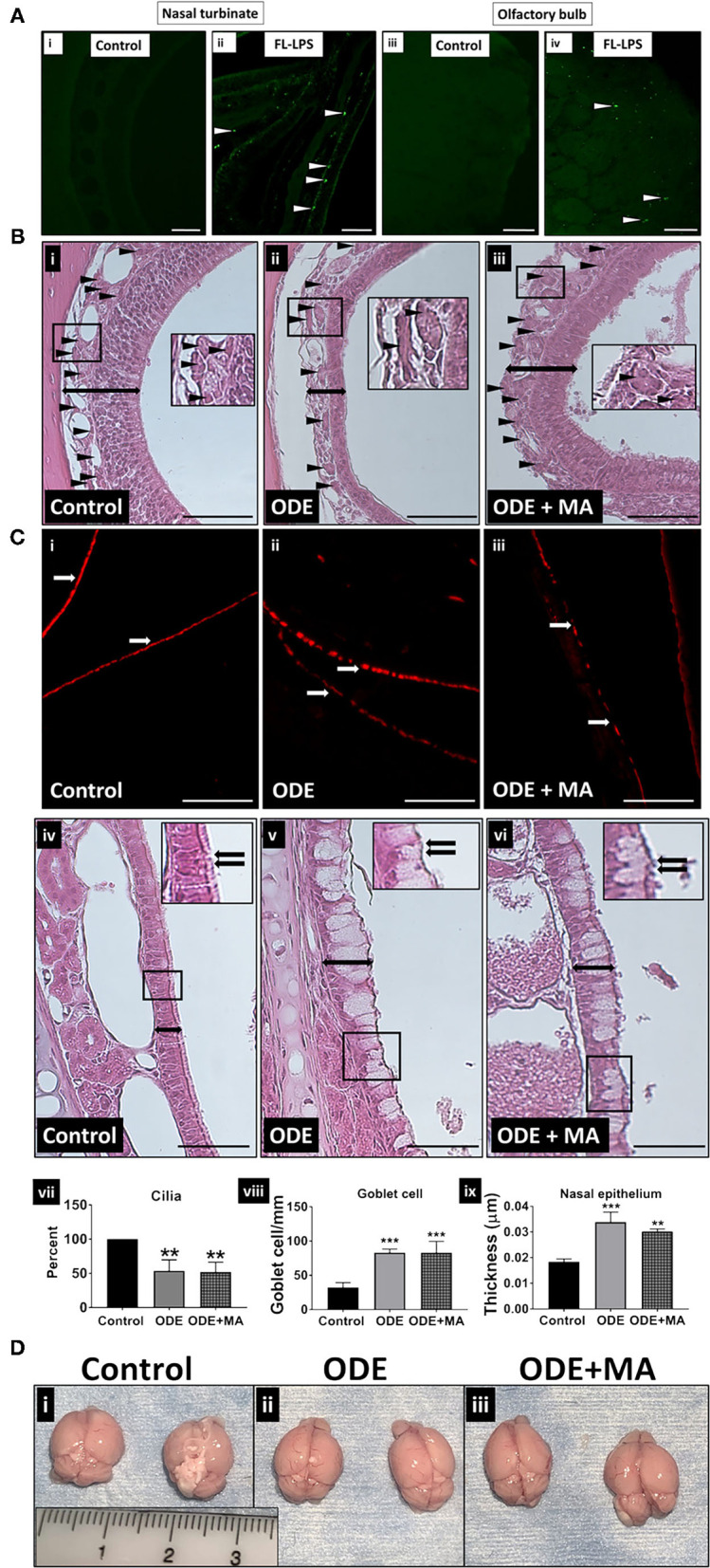
Organic dust extract (ODE) induces gross and histological inflammatory changes in nasal passage and fluorescent lipopolysaccharide (LPS) travel to the olfactory bulb after inhalation. Mice were exposed to various treatments outlined in the Supplementary Table 1. Paraffin embedded sections from perfused (4 paraformaldehyde) mice brain and decalcified upper respiratory tract are shown **(A–C)**. Compared to control, Fluorescent LPS (white arrowhead) was observed in nasal turbinate and the olfactory bulb of the brain (**A**: i–iv, micrometer bar = 100 μm, 20x). When compared to control, mice exposed to ODE showed atrophic changes in the olfactory epithelium (double sided arrow) and fewer nerve bundles (black arrowhead) in nasopharyngeal meatus. Mitoapocynin (MA) treatment partially rescued olfactory epithelium atrophy and nerve bundle loss (**B**: i–iii, micrometer bar = 100 μm, 20x). Immunofluorescent staining of the nasopharyngeal meatus (coronal sections) with acetylated α-tubulin showed a significant loss of cilia (white arrow) in mice treated with ODE (**C**: i–iii, micrometer bar = 100 μm, 20x), and MA treatment had no effect on cilia loss (**C**: vii). Goblet cell numbers and nasal epithelium thickness (two-sided arrow) were analyzed by a blinded investigator (**C**: iv–vi, micrometer bar = 50 μm, 40x). Compared to control, ODE exposure induced goblet cell hyperplasia and thickening of the nasal epithelium, and MA treatment had no effect (**C**: viii, ix). Upon visual gross examination of freshly dissected non-perfused brains, ODE-exposed mice showed signs of congestion when compared to mice administered with normal saline and MA (C11), which appears to reduce congestion in brain (**D**: i–iii).

**Figure 2:**
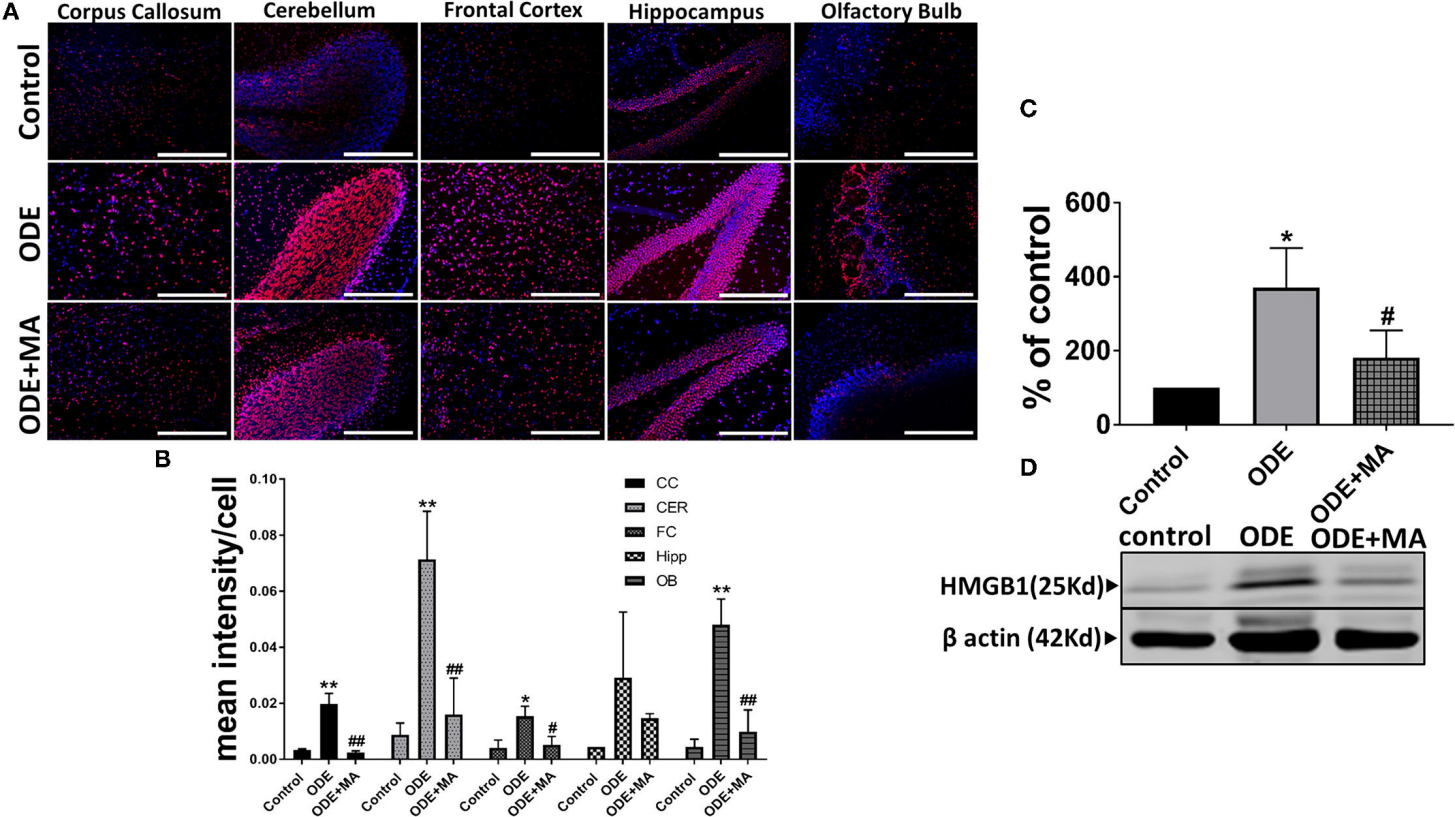
ODE induces HMGB1 expression in various regions of mice brain. Paraffin embedded sections from perfused mice brain were stained with anti-HMGB1 (Cy3, red) antibody and nuclei were identified with 4', 6-Diamidino-2-Phenylindole, Dihydrochloride (DAPI, blue) **(A)**. HMGB1 expression in immunohistochemistry (IHC) was quantified by a blinded investigator. Compared to control, ODE-exposed mice showed higher amounts of HMGB1 staining in the corpus callosum, cerebellum, frontal cortex, and olfactory bulb of the brain. MA (C11) treatment significantly reduced HMGB1 expression **(B)**. Whole cell lysate from freshly dissected mice brain were processed for western blot analysis. HMGB1 and β-actin antibodies (house-keeping protein) detected 25 and 42 kD bands, respectively. Densitometry of normalized bands showed that, compared to controls, ODE exposure increased the HMGB1 protein level in mice brain and that MA treatment significantly reduced HMGB1 protein levels **(C,D)** (*n* = 5, *exposure effect, ** indicates a significant change with respect to control, # MA treatment effect, ## indicates significant change with respect to MA (mitoapocynin). *p* < 0.05, micrometer bar = 50 μm).

**Figure 3:**
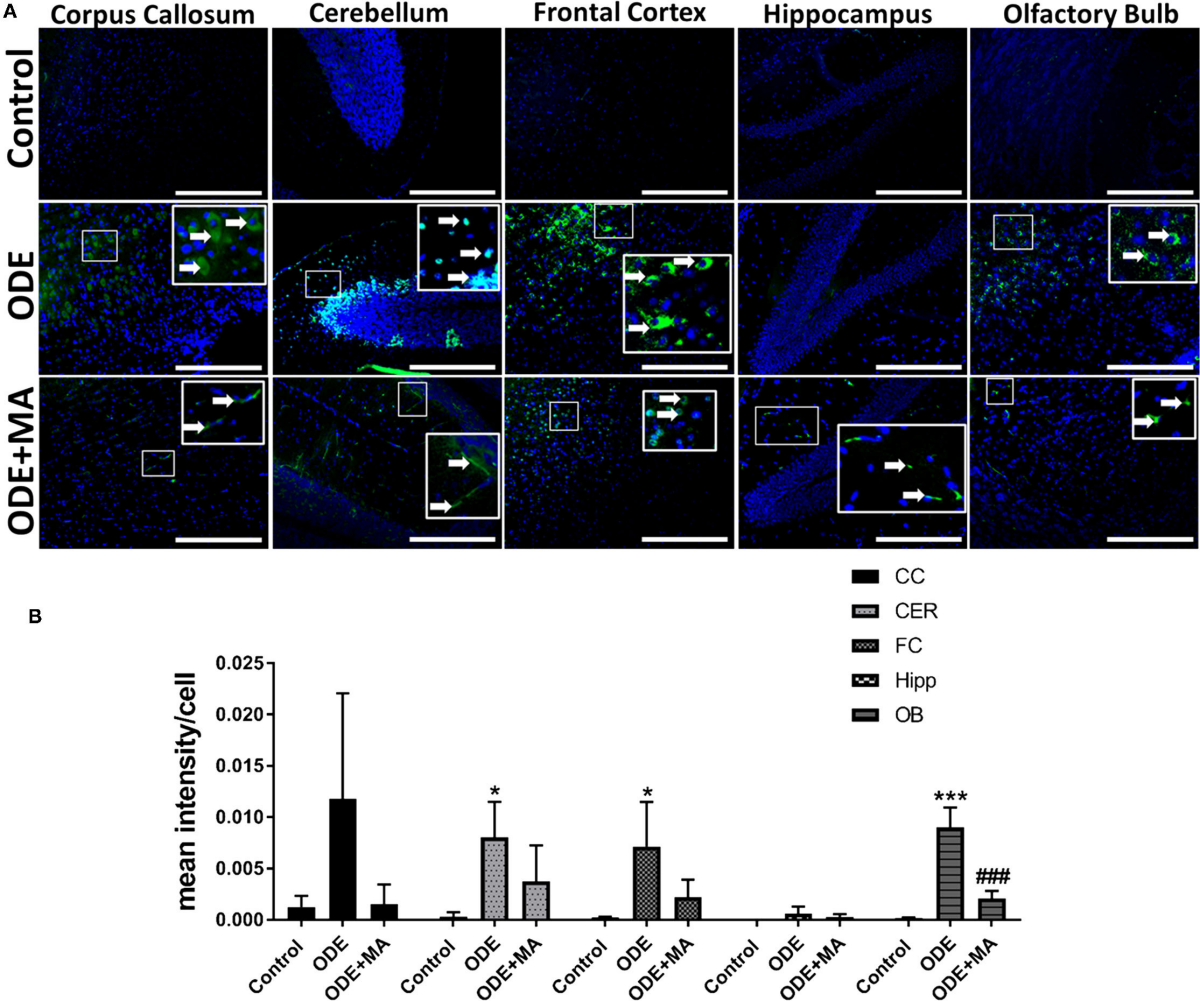
ODE induces 3-Nitrotyrosine generation in various regions of mice brain. Paraffin-embedded sections from perfused mice brain were stained with anti-3-nitrotyrosine (NT; white arrow; Fluorescein isothiocyanate, FITC; green) antibody and nuclei were identified with DAPI (blue) **(A)**. 3-NT generation in IHC was quantified by a blinded investigator. Compared to control, ODE-exposed mice showed higher amounts of 3-NT staining in the cerebellum, frontal cortex, and olfactory bulb of the brain. MA (C11) treatment significantly reduced 3-NT generation only in olfactory bulb area of the brain **(B)** (*n* = 5, *exposure effect, **indicates a significant change with respect to control, # MA treatment effect, ## indicates significant change with respect to MA (mitoapocynin), *p* < 0.05, micrometer bar = 50 μm).

**Figure 4:**
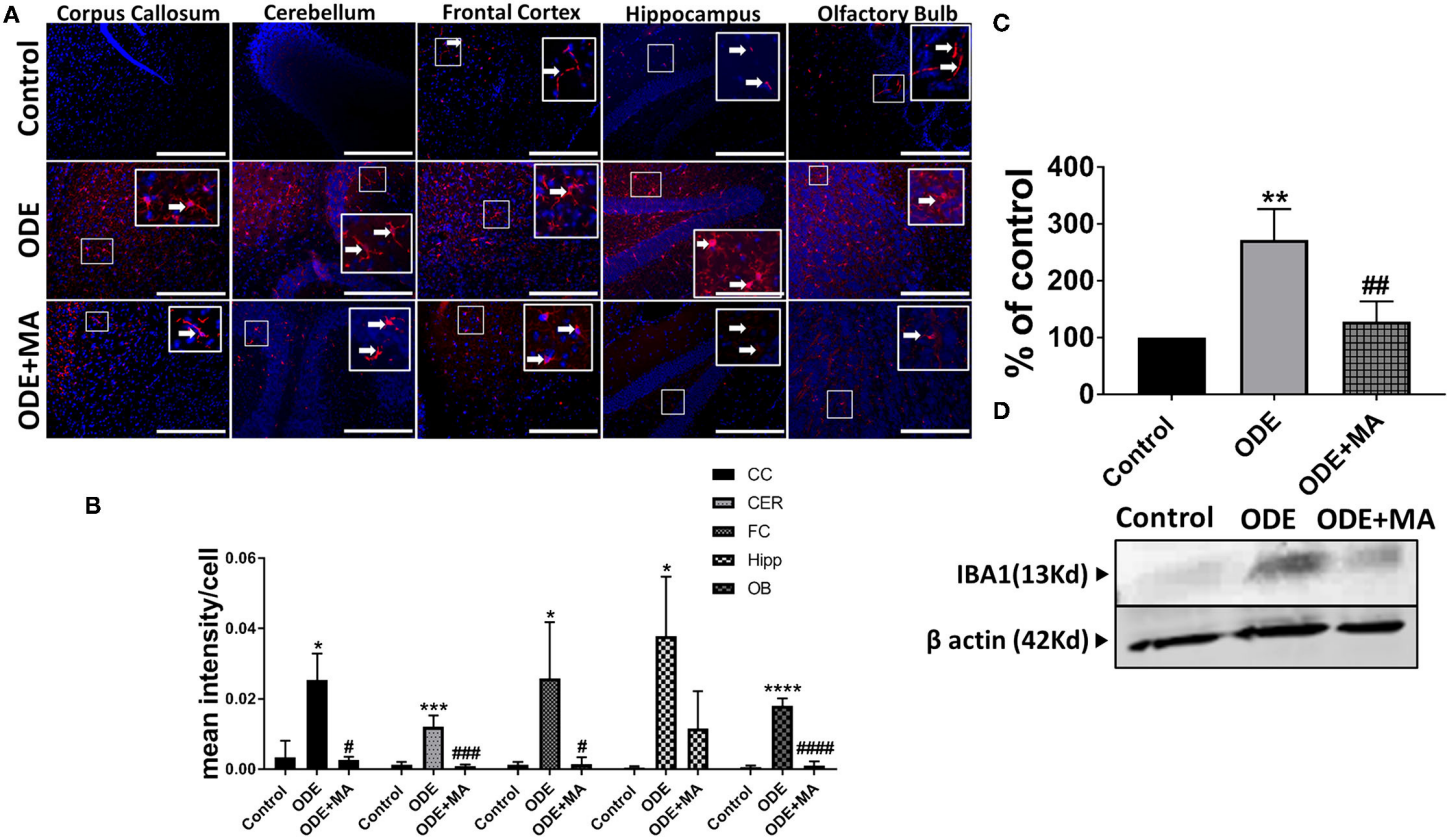
ODE induces IBA1 expression in various regions of mice brain. Paraffin-embedded sections from perfused mice brain were stained with anti-IBA1 (white arrow, Cy3, red) antibody, and nuclei were identified with DAPI (blue). **(A)** IBA1 expression in IHC was quantified by a blinded investigator. Compared to control, ODE-exposed mice showed higher amounts of IBA1 staining in the corpus callosum, cerebellum, frontal cortex, hippocampus, and olfactory bulb of the brain. MA (C11) treatment significantly reduced IBA1 expression in the corpus callosum, cerebellum frontal cortex, and olfactory bulb regions of brain **(B)**. Whole cell lysate from freshly dissected mice brain were processed for western blot analysis. IBA1 and β-actin antibodies (house-keeping protein) detected 13 and 42 kD bands, respectively. Densitometry of normalized bands showed that, compared to controls, ODE exposure increased the IBA1 protein level in mice brain, and that MA treatment significantly reduced IBA1 protein level **(C,D)** (*n* = 5, *exposure effect, # MA treatment effect, *p* < 0.05, micrometer bar = 50 μm).

**Figure 5:**
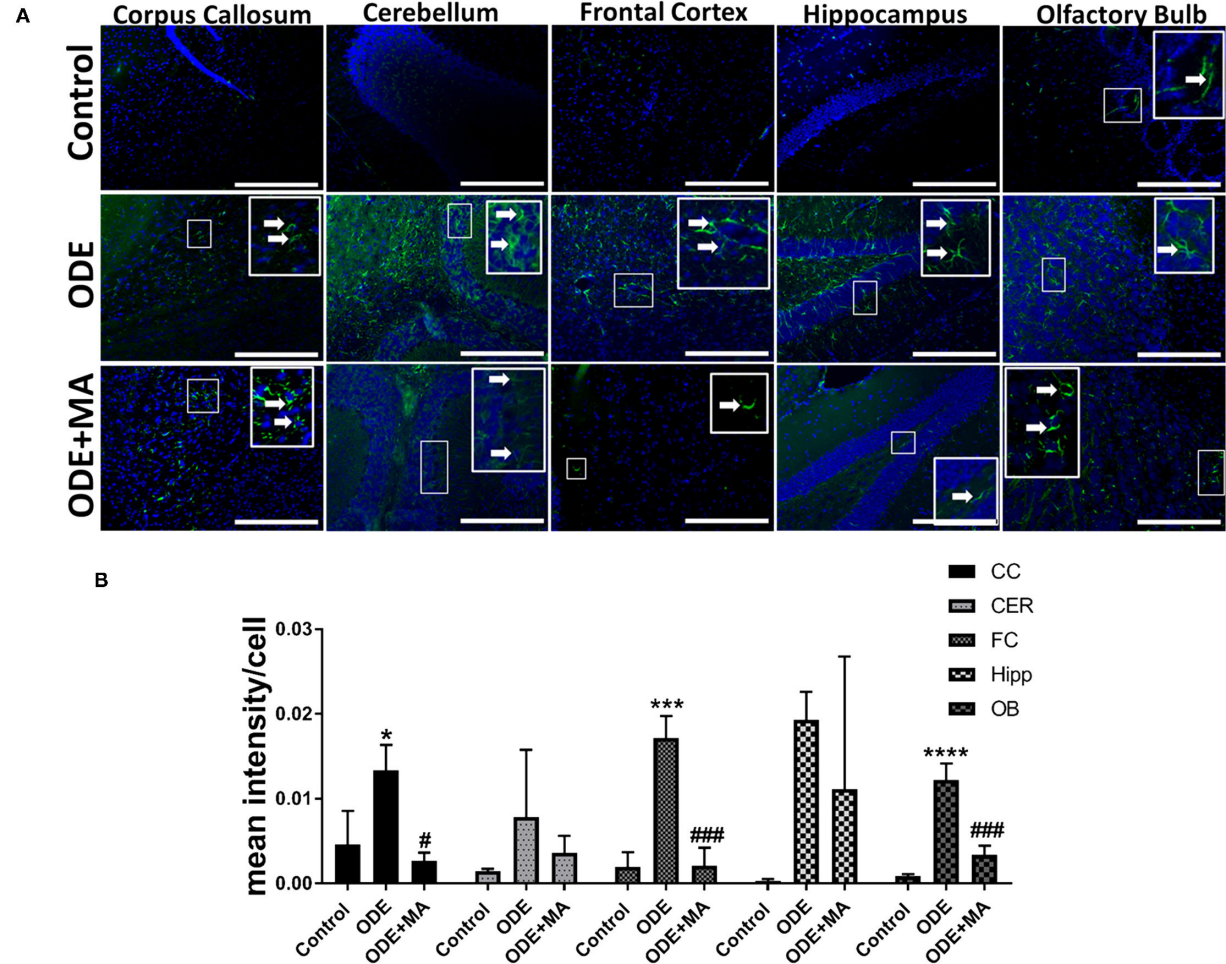
ODE induces glial fibrillary acidic protein (GFAP) expression in various regions of mice brain. Paraffin-embedded sections from perfused mice brain were stained with anti-GFAP (white arrow, FITC, green) antibody and nuclei were identified with DAPI (blue) **(A)**. GFAP expression in IHC was quantified by a blinded investigator. Compared to control, ODE-exposed mice showed higher amounts of GFAP staining in the corpus callosum, frontal cortex, and olfactory bulb of the brain. MA (C11) treatment significantly reduced GFAP expression in the corpus callosum, frontal cortex, and olfactory bulb regions of brain **(B)** (*n* = 5, *exposure effect, # MA treatment effect, *p* < 0.05, micrometer bar = 50 μm).

**Figure 6:**
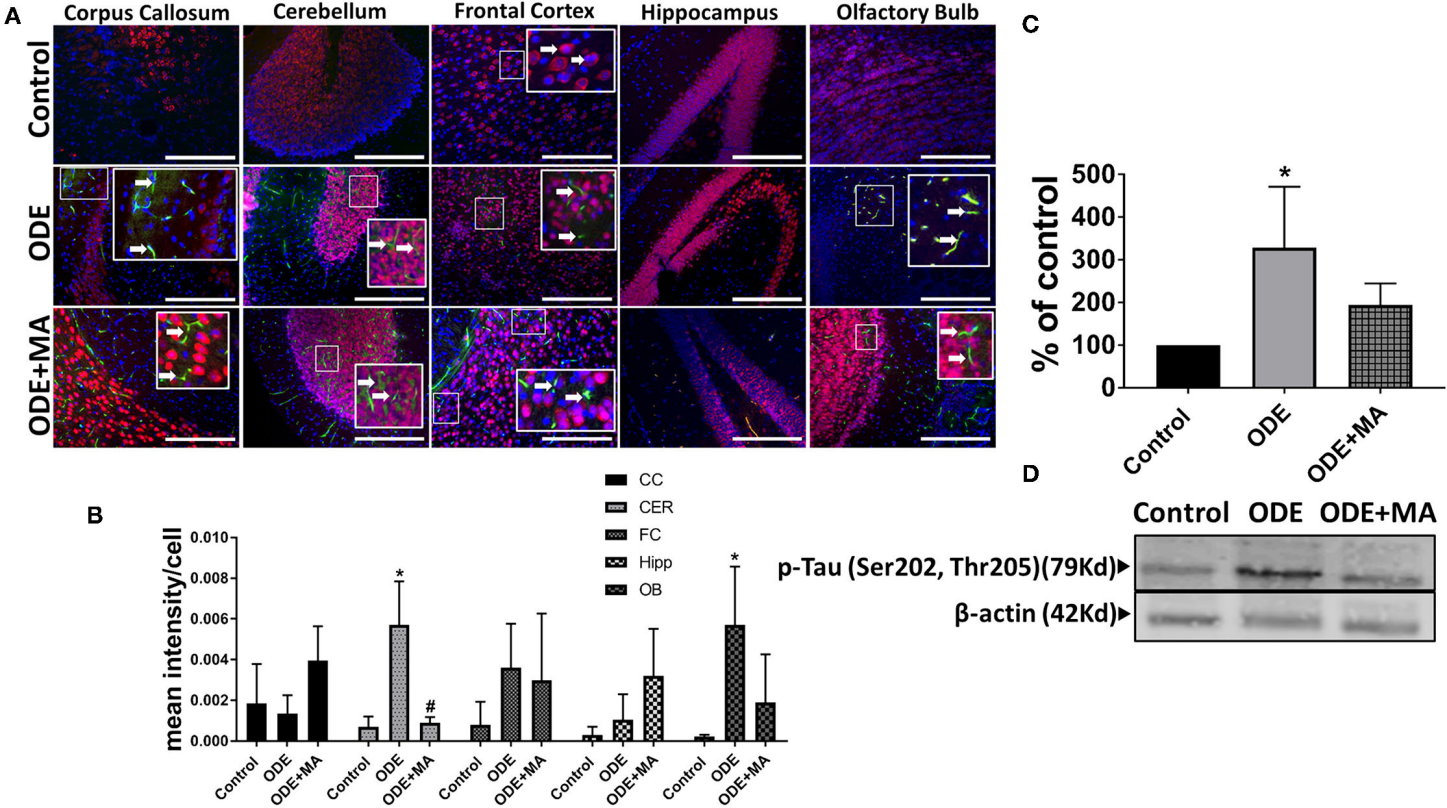
ODE induces hyperphosphorylated Tau (p-Tau) expression in various regions of mice brain. Paraffin-embedded sections from perfused mice brain were stained with anti p-Tau (white arrow, FITC, green) and anti-NeuN (neuronal marker; Cy3, red) antibodies. Also, nuclei were identified with DAPI (blue) **(A)**. p-Tau expression in IHC was quantified by a blinded investigator. Compared to control, ODE-exposed mice showed higher amounts of p-Tau staining in the cerebellum and olfactory bulb of the brain. MA (C11) treatment significantly reduced p-Tau expression in the cerebellum region of brain **(B)**. Whole cell lysate from freshly dissected mice brain were processed for western blot analysis. p-Tau and β-actin antibodies (house-keeping protein) detected 79 and 42 kD bands, respectively. Densitometry of normalized bands showed that, compared to controls, ODE exposure increased the p-Tau protein level in mice brain and MA treatment significantly reduced p-Tau protein level **(C,D)** (*n* = 5, *exposure effect, # MA treatment effect, *p* < 0.05, micrometer bar = 50 μm).

**Figure 7:**
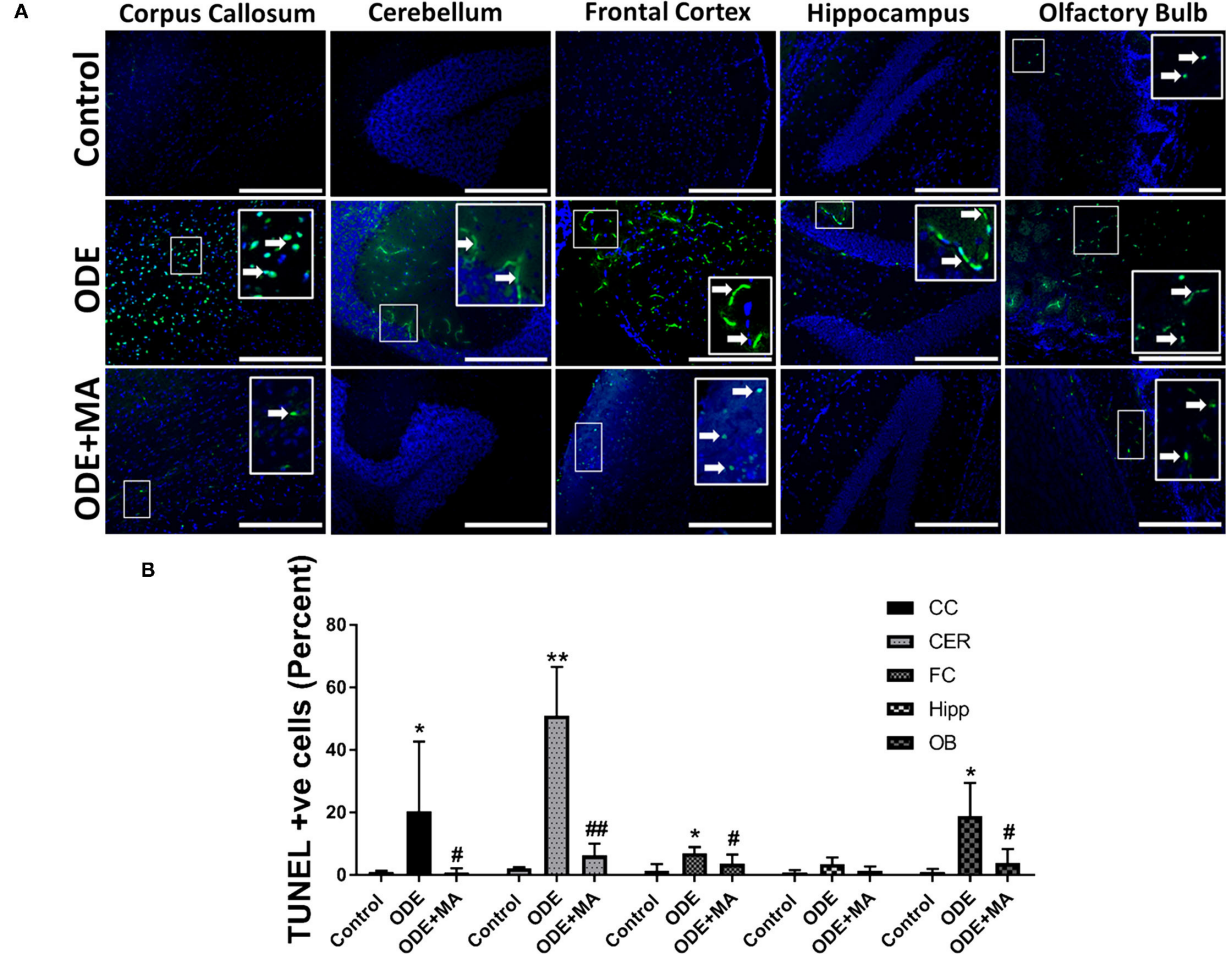
ODE exposure induces neurodegenerative changes in various regions of mice brain. Paraffin-embedded sections from perfused mice brain were labeled with deoxyuridine triphosphate (dUTP)-FITC (white arrow; apoptosis marker, FITC, green), and nucleus was stained with DAPI (blue) **(A)**. The total number of cells (DAPI, blue) and terminal deoxynucleotidyl transferase dUTP nick end labeling (TUNEL) positive cells (FITC, green) per field (20X) were counted in a total of five random fields by a blinded investigator. Compared to control, ODE-exposed mice showed a higher number of TUNEL positive cells in the corpus callosum, cerebellum, frontal cortex, and olfactory bulb of the brain. MA (C11) significantly reduced the number of TUNEL positive cells in the corpus callosum, cerebellum, frontal cortex, and olfactory bulb regions of the mice brain **(B)** [*n* = 5, *exposure effect, # MA (C11) treatment effect, *p* < 0.05, micrometer bar = 50 μm].

**Figure 8:**
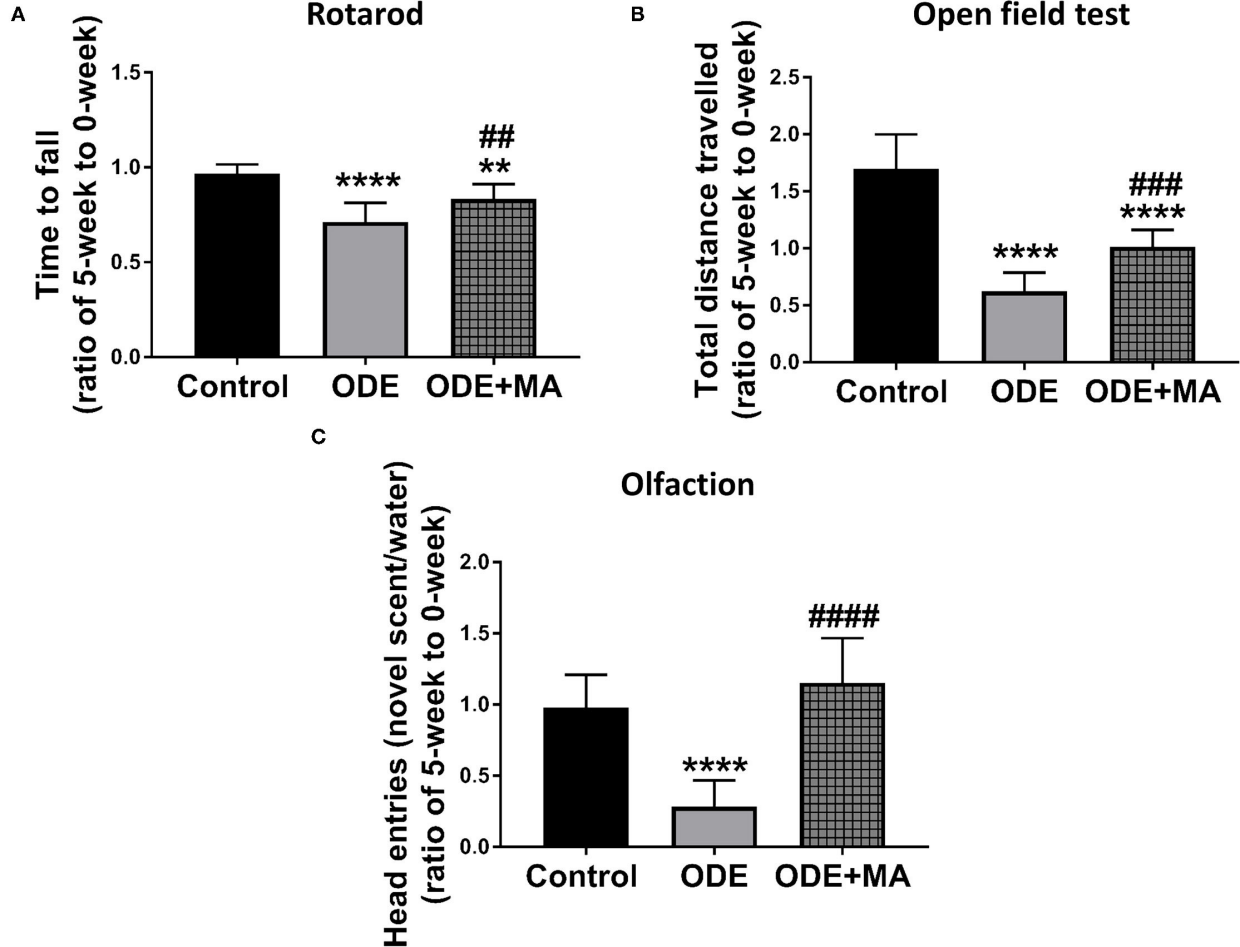
ODE exposure induces neuro-motor and neuro-sensory changes in mice. As compared to control, ODE-exposed mice displayed a significant decrease in the time to fall from the rotarod in an accelerated profile, whereas mice administered with oral dosages of MA (C11) showed a significant higher time to fall from the rotarod when compared to ODE-exposed group **(A)**. As compared to control, ODE-exposed mice showed significantly lower exploratory locomotor activity. Whereas, mice administered with oral dosages of MA(C11) showed a significant higher exploratory locomotor activity when compared to ODE-exposed **(B)**. As compared to control, ODE exposed mice showed a significantly lower ability of olfactory sensation and mice administered with MA (C11) showed a significant higher sense of olfactory ability when compared to mice exposed to only ODE **(C)**.

All *in vivo* data were determined from 11 to 12 biological replicates (*n* = 11–12 mice/group), with three technical replicates/mouse. Data were expressed as mean ± SEM and analyzed by *T*-test or one-way or two-way ANOVA followed by Bonferroni's *post hoc* comparison tests (GraphPad Prism 5.0, La Jolla, California). A *p* ≤ 0.05 was considered statistically significant. An asterisk (^*^) indicates a significant difference between controls and ODE-treated cells, whereas hashtag (#) indicates MA treatment. The *p*-values (Figures 1–**8**) corresponding to asterisk/s or hashtag/s are listed in Supplementary Table 4.

## Results

### ODE Exposure Induces Microscopic and Gross Changes in the Nasal Epithelium and Brain, Respectively

Using bright field and fluorescent microscopy, we assessed histological changes in the nasal epithelium and brain tissue, respectively. Compared to control, 5-weeks of intranasal administration of ODE and FL-LPS induced inflammatory changes. Upon examination under a fluorescent microscope (FITC lens), we observed FL-LPS (tagged with FITC) particles in the nasal epithelium and the olfactory bulb of the brain (Figures 1Ai–iv). Light microscopic examination of the upper respiratory tract sections from the mice treated with ODE revealed atrophic changes in the olfactory epithelium with significant loss of olfactory nerve bundles. Mice exposed to ODE with MA treatment showed a partial protection indicated by a reduction in the atrophic changes and loss of nerve bundles in the olfactory epithelium. Atrophic changes in the olfactory epithelium and reduction in the number of olfactory nerve bundles are suggestive of hyposmia (Figure 1Bi–iii). Immunostaining of nasopharyngeal meatus of mice exposed to ODE also indicated a progressive loss of cilia lining. MA treatment of mice did not prevent the loss of cilia (Figure 1Ci–iii,vii). Significant goblet cell hyperplasia and thickening of nasal epithelium was observed in mice exposed to ODE for 5-weeks, indicative of chronic inflammation of the nasal mucosa (Figure 1Civ–vi,viii,ix). MA treatment did not show any significant effect on ODE-induced nasal epithelium changes (Figure 1Cviii,ix). Upon gross visual examination, ODE-exposed mice brain showed signs of significant congestion when compared to mice exposed to normal saline (control). MA (C11)-administered mice appeared to have less congestion in the brain (Figure 1Di–iii).

### ODE Exposure of Mice and HMGB1 Expression in Brain

Immunohistochemical expression of HMGB1 in control (saline treated) and ODE-treated mice is shown in Figure 2A. Quantification of IHC data showed that, compared to controls, ODE increased the expression of HMGB1 in the corpus callosum, cerebellum, frontal cortex, and olfactory bulb regions of the brain. MA(C11) treatment significantly decreased ODE-induced increases in HMGB1 expression (Figure 2B). Western blotting on whole tissue lysates showed that, compared to controls, ODE increased the expression of HMGB1 after 5-week exposure, whereas MA(C11) treatment significantly decreased the same (Figures 2C,D).

### ODE Exposure of Mice and 3-Nitrotyrosine (3-NT) Generation in the Brain

Immunohistochemical expression of 3-nitrotyrosine (NT) in control (saline-treated) and ODE-treated mice is shown in Figure 3A. Quantification of intraclass correlation coefficient (ICC) data showed that, compared to controls, ODE exposure increased the generation of 3-NT in the cerebellum, frontal cortex, and olfactory bulb regions of the brain. MA(C11) treatment significantly decreased 3-NT generation in olfactory bulb region of the ODE-exposed mice (Figure 3B).

### ODE Exposure of Mice and Microglial Activation in Brain

Immunohistochemical expression of IBA1 in control (saline-treated) and ODE-treated mice is shown in Figure 4A. Quantification of ICC data showed that, compared to controls, ODE increased the expression of IBA1 in the corpus callosum, cerebellum, frontal cortex, hippocampus, and olfactory bulb regions of the brain. MA(C11) treatment significantly decreased ODE-induced increases in IBA1 expression in the corpus callosum, cerebellum, frontal cortex, and olfactory bulb (Figure 4B). Western blotting on whole tissue lysates showed that, compared to controls, ODE increased the expression of IBA1 after 5-week exposure, whereas MA(C11) treatment significantly decreased the same (Figures 4C,D).

### ODE Exposure of Mice and Astroglial Activation in the Brain

Immunohistochemical expression of GFAP in control (saline-treated) and ODE-treated mice is shown in Figure 5A. Quantification of ICC data showed that, compared to controls, ODE increased the expression of GFAP in the corpus callosum, frontal cortex, and olfactory bulb regions of the brain. MA(C11) treatment significantly decreased GFAP expression in the corpus callosum, frontal cortex, and olfactory bulb region of the ODE-exposed mice (Figure 5B).

### ODE Exposure of Mice and p-Tau Expression in Brain

Immunohistochemical expression of p-Tau in control (saline treated) and ODE-treated mice is shown in Figure 6A. Quantification of ICC data showed that, compared to controls, ODE increased the expression of p-Tau in the cerebellum and olfactory bulb regions of the brain. MA(C11) treatment significantly decreased ODE-induced increases in IBA1 expression in cerebellum (Figure 6B). Western blotting on whole tissue lysates showed that, compared to controls, ODE increased the expression of p-Tau after 5-week exposure, whereas MA(C11) treatment significantly decreased the same (Figures 6C,D).

### ODE Exposure Induced Apoptosis in Mice Brain

Fluorescent TUNEL staining revealed that, compared to control, ODE-exposed mice showed a significantly higher number of degenerating or dead cells in the corpus callosum, cerebellum, frontal cortex, and olfactory bulb regions of the mice brain. MA(C11) significantly reduced the number of TUNEL positive cells in the corpus callosum, cerebellum, frontal cortex, and olfactory bulb when compared to ODE treated mice (Figures 7A,B).

### ODE Exposure and Neurobehavior Changes in Mice

After mice were exposed to either normal saline or ODE with or without oral MA(C11), behavioral and olfactory experiments were performed. Five weeks of ODE treatment induced a locomotor deficit in mice, indicated by deficits in rotarod and open field test. MA(C11) oral administration significantly restored the locomotor activity after 5-weeks when compared to ODE-treated mice (Figures 8A,B). Following a 5-week exposure to ODE, sense of olfaction was significantly reduced, and oral administration of MA(C11) significantly restored the olfactory ability of mice after 5-weeks when compared to ODE treated mice (Figure 8C).

## Discussion

Exposure to contaminants have been shown to be a major etiology of neurodegenerative diseases with an underlying inflammatory process (Molot et al., 2021). Higher prevalence of neurodegenerative disorders, such as PD and AD in people living in the Midwestern and northeastern parts of the USA, is concerning due to a possible link to persistent exposure to agriculture contaminants (Wright Willis et al., 2010; U.S. Geological Survey, 2021). Overall, data contained in this manuscript confirm that inhaled OD induces neuroinflammation which results in neurodegeneration and sensory-motor deficits. Further, mitochondria-targeting NOX-2 inhibition using MA is a promising therapy.

In animal production units, workers are exposed to contaminants including OD via inhalation route (Nordgren and Charavaryamath, 2018). However, our current study uses an intra-nasal instillation of ODE into mouse nostrils (Poole et al., 2009; Shrestha et al., 2021). However, this model is well-established and is known to recapitulate many features of inhalational exposure to the animal production units (Charavaryamath et al., 2008; Poole et al., 2009). We have previously used inhalational models of rat (Charavaryamath et al., 2005) and mouse (Charavaryamath et al., 2008) models that mimicked human occupational exposure to organic dust exposure. Others have reviewed the use of farm animals (McClendon et al., 2015), rabbits, and guinea pigs (Donham and Leininger, 1984) to study ODE.

We followed a published protocol (Hanson et al., 2013) of intranasal administration to preferentially target delivery to the brain. Our data showed that inhaled FL-LPS is localized in the nasal turbinate and olfactory bulb, indicating that inhaled ODE may possibly take a similar route. This assumption is made because ODE contains significant amount of LPS. However, commercial FL-LPS and ODE may not have similar dosimetry as the particle size and surface charges, and other properties could be different. Regardless, in general, majority of the changes due to inhaled ODE were localized to the olfactory bulb, and exposed mice showed a compromise in their olfaction ability. Hence, inhaled ODE may be using olfactory axons as the Trojan horses to reach and accumulate in the olfactory bulbs (Lucchini et al., 2012). Our intra-nasal administration volume exceeds that of the nasal cavity of an 8-week-old C57BL6 mouse. Hence, the volume may be swallowed and/or inhaled, leading to the uptake via olfactory and/or trigeminal pathways (Maigler et al., 2021). We also do not rule out the possibility of systemic route of ODE-exposure-induced neuroinflammation via lung-gut-brain axis (Crawford et al., 2021).

Liposaccharide is one of major PAMP present in the ODE, and our data confirms that inhaled ODE induced atrophic changes in the olfactory epithelium with loss of olfactory nerve bundles. Further, a 5-week exposure induced loss of cilia and increase in goblet cell numbers to indicate a compromise in innate immunity in the local upper respiratory tract. Orally administered MA was partially protective to underscore the importance of mitochondrial targeting therapy. Interestingly, behavioral studies in mice showed that exposed mice had a decreased ability of olfaction, and that MA therapy significantly restored the ability to smell. Therefore, despite a partial protective effect at the histological levels, mice showed full recovery as per the results of the functional assays. Several of these changes seen in our model are typical findings in cigarette smokers (Leopold et al., 2009; Cao et al., 2018).

Our visual examination of brains confirmed that ODE exposure induced a congestion, indicating an inflammatory process and that MA treatment reduced these lesions to indicate a general anti-inflammatory effect. Next, our data showed a general increase in the levels of HMGB1 in the brains of the exposed animals. In addition, MA therapy decreased the levels of HMGB1. This data highlight a possible role for HMGB1, namely, a DAMP (Ugrinova and Pasheva, 2017) in ODE-induced neuroinflammation. Further, we demonstrated that cytoplasmic NOX-2 inhibition is effective in reducing the levels of HMGB1 and in further reducing a vicious cycle of inflammation. These observations are in line with our previous work wherein we demonstrated how HMGB1 could be targeted to reduce ODE-induced respiratory (Bhat et al., 2019) and neuroinflammation (Massey et al., 2019).

In our 5-week OD exposure model, we observed that exposure induced the activation of microglia (increase in IBA1) (Hovens et al., 2014), glial cell activation (increase in GFAP) (Eng et al., 1992; Dong and Benveniste, 2001), and 3-NT production (marker of neuroinflammation), and also the significant increase in the expression of phosphorylated-Tau protein (ser202, thr205) in the cerebellum and olfactory bulbs to highlight neuroinflammatory process primarily driven by microglia and astrocytes with overwhelming oxidative stress. In general, oral MA treatment decreased exposure-induced HMGB1, IBA1, and GFAP without any effect on phosphorylated Tau expression levels.

3-nitrotyrosine is a product of tyrosine following nitration, with peroxynitrite and peroxynitrite formed from nitric oxide and superoxide (Ohmori and Kanayama, 2005; Sarkar et al., 2020). Following ODE exposure, the 3-NT generation in different regions of the brain was found to be significantly increased, indicating active neuroinflammation. MA(C11) is a cytoplasmic NOX-2 inhibitor and can result in the inhibition of superoxide generation. As expected, the oral treatment of MA(C11) decreased 3-NT expression in different regions of mice brain exposed to OD treatment.

IBA1 is a protein specifically expressed in microglia and is upregulated during microglial activation (Hovens et al., 2014). IBA1 expression was found to be elevated in different regions of the brains of mice after 5-weeks of ODE exposure. Mice given oral treatment of MA(C11) in conjunction with ODE exposure showed significantly less IBA1 in mice brains. This finding is crucial in the sense that intranasal ODE exposure, in an *in viv*o setting, is able to induce immune cell activation that is very similar to *in vitro* and *ex vivo* models (Massey et al., 2019).

Glial fibrillary acidic protein in the CNS is expressed in astrocyte cells. Astrocytes are involved in cell communication and in repair after injury to the brain. Astrocytes are also immunocompetent in nature and can react to neurodegenerative insults, rapidly leading to vigorous astrogliosis (Eng et al., 1992; Dong and Benveniste, 2001). However, activated astrocytes secrete various neurotrophic factors for neuron survival. A rapid and large-scale astroglia activation has also been known to exacerbate neuroinflammation (Tani et al., 1996). GFAP expression was found to be significantly upregulated in mice brain upon 5-week ODE exposure. MA(C11) significantly reduced GFAP expression in various regions of brain upon oral treatment. This wide scale GFAP expression in the mice brain is in response to an ongoing neurodegenerative process in the brain.

Tau proteins are a group of six highly soluble proteins isoforms that help in maintaining the microtubules in axons of neurons in the CNS. p-Tau is an insoluble aggregates of Tau and is associated with neurocognitive disorders like PD and AD (Iqbal et al., 2010). After 5-week intranasal exposure with ODE, mice brains showed aggregates of p-Tau in the axons of neurons. This clearly indicates that continued ODE exposure can predispose the brain to neurocognitive disorders. MA(C11) oral treatment was able to reduce overall p-Tau expression only in the cerebellum, indicating a partial protection against ODE-induced cognitive deficit.

Neuroinflammation is almost always accompanied by neurodegeneration (Ransohoff, 2016), and is possibly due to the activation of microglia and astroglia, which can lead to neuronal death (Tani et al., 1996; Sarkar et al., 2020). In this study, we employed TUNEL assay to label apoptotic cell in the brain following a 5-week ODE exposure. TUNEL positive cells were found in the brain after ODE administration, and oral administration of MA(C11) alleviated neurodegeneration by reducing the number of TUNEL positive cells. These results are in agreement with our previous findings from our *in vitro* and *ex vivo* models (Massey et al., 2021a).

Motor and neurosensory deficits are generally associated with neurocognitive disorders (Nuber et al., 2008; Seo et al., 2018). Recent evidence have shown that the olfactory dysfunction (hyposmia) is the first sign of neurodegeneration, thus making it a key marker of an early of neurological disorders (Emanuele and Lavinia, 2018; Seo et al., 2018). Mice, after 5-weeks of ODE exposure, developed signs of neuro-motor deficit that were indicated by decrease in fall time (time taken to fall) on the rotarod. Mice also showed a significant decrease in the overall locomotor activity after exposure to ODE. A significant hyposmia was also observed in mice upon ODE exposure. Mice that were administered with MA(C11) together with ODE showed significant improvement in neuro-motor deficit, locomotor activity, and olfactory ability.

Despite the above findings, we observed a reduction in locomotor activity of mice upon OD exposure. In addition, we did not perform any behavioral analysis for depression. A very limited neurochemical analysis (Supplementary Figure 1) was performed, which was indicative of signs of depression, but a thorough investigation is highly desired to explore the role of OD in inducing depression. A complete RNA sequencing of isolated microglia from the brain of mice may provide genomic insights into how the ODE exposure drives neuroinflammation. Analyzing changes at the gene level could very well lead to a good understanding of microglial activation and further help in developing a robust therapeutic strategy to abrogate OD exposure-induced neuroinflammation.

## Conclusion

In conclusion, based on the detection of inhaled fluorescent LPS in the olfactory bulb, it is likely that even inhaled OD has the potential to reach the brain directly via the olfactory nerve axons and invoke an inflammatory response. Following a five exposure to ODE, atrophy of the olfactory epithelium, pathologies in the olfactory bulb, p-Tau aggregation, and neuro-motor deficits were documented, indicating an early onset of neurocognitive disorders. MA therapy appears to be partially protective against the ODE induced changes, thus leaving opportunities for developing new therapeutic strategies.

## Data Availability Statement

The original contributions presented in the study are included in the article/Supplementary Material, further inquiries can be directed to the corresponding authors.

## Ethics Statement

The animal study was reviewed and approved by Institutional Animal Care and Use Committee (IACUC) protocols at the Iowa State University.

## Author Contributions

NM participated in the design of the experiments, conducted majority of the experiments, analyzed the data, and drafted the manuscript. DS and SB helped and coordinated with the experiments and edited the manuscript. PP performed the neurochemical analysis, acquired the data, and participated in writing the manuscript. CW helped with the design of the experiments and statistical analysis of the data. LK collected the organic dust samples, transported, and edited the manuscript. JS helped in designing the brain and upper respiratory tract tissue collection plan, performed blinded tissue pathology analysis, acquired the images, and edited the manuscript. AK and CC were involved in the design of the experiments, participated in the interpretation of the data, edited the manuscript, and provided funding support. All authors agree to be accountable for the content of the work and approved the final manuscript.

## Funding

CC laboratory was funded through startup grant through Iowa State University and a pilot grant (5 U54 OH007548) from the Centers for Disease Control and Prevention-The National Institute for Occupational Safety and Health (CDC-NIOSH). AK laboratory was supported by the National Institutes of Health grants (ES026892, ES027245, and NS100090).

## Conflict of Interest

AK is a shareholder of PK Biosciences Corporation Ames, IA, which is interested in identifying novel biomarkers and potential therapeutic targets for Parkinson's disease. The remaining authors declare that they have no potential conflicts of interest with respect to the research, authorship, and/or publication of this article.

## Publisher's Note

All claims expressed in this article are solely those of the authors and do not necessarily represent those of their affiliated organizations, or those of the publisher, the editors and the reviewers. Any product that may be evaluated in this article, or claim that may be made by its manufacturer, is not guaranteed or endorsed by the publisher.

## References

[B1] Bhat S. M. Massey N. Karriker L. A. Singh B. Charavaryamath C. (2019). Ethyl pyruvate reduces organic dust-induced airway inflammation by targeting HMGB1-RAGE signaling. Respir. Res. 20:27. 10.1186/s12931-019-0992-3 30728013 PMC6364446

[B2] Cacciottolo M. Wang X. Driscoll I. Woodward N. Saffari A. Reyes J.. (2017). Particulate air pollutants, APOE alleles and their contributions to cognitive impairment in older women and to amyloidogenesis in experimental models. Transl. Psychiatry. 7, e1022. 10.1038/tp.2016.280 28140404 PMC5299391

[B3] Calderón-Garcidueñas L. Kavanaugh M. Block M. D'Angiulli A. Delgado-Chávez R. Torres-Jardón R.. (2012). Neuroinflammation, hyperphosphorylated tau, diffuse amyloid plaques, and down-regulation of the cellular prion protein in air pollution exposed children and young adults. J. Alzheimer's Dis. 28, 93–107. 10.3233/JAD-2011-110722 21955814

[B4] Cao X. Wang Y. Xiong R. Muskhelishvili L. Davis K. Richter P. A.. (2018). Cigarette whole smoke solutions disturb mucin homeostasis in a human *in vitro* airway tissue model. Toxicology 409, 119–128. 10.1016/j.tox.2018.07.015 30053496

[B5] Charavaryamath C. Janardhan K. S. Townsend H. G. Willson P. Singh B. (2005). Multiple exposures to swine barn air induce lung inflammation and airway hyper-responsiveness. Respir. Res. 6:50. 10.1186/1465-9921-6-50 15932644 PMC1164433

[B6] Charavaryamath C. Juneau V. Suri S. S. Janardhan K. S. Townsend H. Singh B. (2008). Role of Toll-like receptor 4 in lung inflammation following exposure to swine barn air. Exp. Lung Res. 34, 19–35. 10.1080/01902140701807779 18205075

[B7] Charavaryamath C. Singh B. (2006). Pulmonary effects of exposure to pig barn air. J. Occup. Med. Toxicol. 1, 10. 10.1186/1745-6673-1-10 16756675 PMC1524789

[B8] Chen J. C. Wang X. Serre M. Cen S. Franklin M. Espeland M. (2017). Particulate air pollutants, brain structure, and neurocognitive disorders in older women. Res. Rep. Health Eff. Inst. 1–65. PMC7266369 31898881

[B9] Crawford M. S. Nordgren T. M. McCole D. F. (2021). Every breath you take: impacts of environmental dust exposure on intestinal barrier function–from the gut-lung axis to COVID-19. Am. J. Physiol. Gastrointestinal. Liver Physiol. 320, G586–G600. 10.1152/ajpgi.00423.2020 33501887 PMC8054554

[B10] Dong Y. Benveniste E. N. (2001). Immune function of astrocytes. Glia 36, 180–190. 10.1002/glia.1107 11596126

[B11] Donham K. J. Leininger J. R. (1984). Animal studies of potential chronic lung disease of workers in swine confinement buildings. Am. J. Vet. Res. 45, 926–931. 6732025

[B12] Dranka B. P. Gifford A. McAllister D. Zielonka J. Joseph J. O'Hara C. L.. (2014). A novel mitochondrially-targeted apocynin derivative prevents hyposmia and loss of motor function in the leucine-rich repeat kinase 2 (LRRK2(R1441G)) transgenic mouse model of Parkinson's disease. Neurosci. Lett. 583, 159–164. 10.1016/j.neulet.2014.09.042 25263790 PMC4253647

[B13] Ehsanifar M. Tameh A. A. Farzadkia M. Kalantari R. R. Zavareh M. S. Nikzaad H.. (2019). Exposure to nanoscale diesel exhaust particles: Oxidative stress, neuroinflammation, anxiety and depression on adult male mice. Ecotoxicol. Environ. Saf. 168, 338–347. 10.1016/j.ecoenv.2018.10.090 30391838

[B14] Emanuele B. Lavinia A. (2018). Olfaction Among the First Senses to Develop and Decline.

[B15] Eng L. F. Yu A. C. Lee Y. L. (1992). Astrocytic response to injury. Prog. Brain Res. 94, 353–365. 10.1016/S0079-6123(08)61764-1 1337615

[B16] Fordyce C. B. Jagasia R. Zhu X. Schlichter L. C. (2005). Microglia Kv1.3 channels contribute to their ability to kill neurons. J. Neurosci. 25, 7139. 10.1523/JNEUROSCI.1251-05.2005 16079396 PMC6725234

[B17] Ghosh A. Chandran K. Kalivendi S. V. Joseph J. Antholine W. E. Hillard C. J.. (2010). Neuroprotection by a mitochondria-targeted drug in a Parkinson's disease model. Free Radic. Biol. Med. 49, 1674–1684. 10.1016/j.freeradbiomed.2010.08.028 20828611 PMC4020411

[B18] Ghosh A. Langley M. R. Harischandra D. S. Neal M. L. Jin H. Anantharam V.. (2016). Mitoapocynin treatment protects against neuroinflammation and dopaminergic neurodegeneration in a preclinical animal model of parkinson's disease. J. Neuroimmun. Pharmacol. 11, 259–278. 10.1007/s11481-016-9650-4 26838361 PMC4995106

[B19] Ghosh A. Saminathan H. Kanthasamy A. Anantharam V. Jin H. Sondarva G.. (2013). The peptidyl-prolyl isomerase Pin1 up-regulation and proapoptotic function in dopaminergic neurons: relevance to the pathogenesis of Parkinson disease. J. Biol. Chem. 288, 21955–21971. 10.1074/jbc.M112.444224 23754278 PMC3724650

[B20] Hanson L. R. Fine J. M. Svitak A. L. Faltesek K. A. (2013). Intranasal administration of CNS therapeutics to awake mice. J. Visualized Exp. 74:4440. 10.3791/4440 23608783 PMC3653240

[B21] Hovens I. B. Nyakas C. Schoemaker R. G. (2014). A novel method for evaluating microglial activation using ionized calcium-binding adaptor protein-1 staining: cell body to cell size ratio. Neuroimmunol. Neuroinflammat. 1, 82–88. 10.4103/2347-8659.139719

[B22] International Labor Organization. (2018). Agriculture: A Hazardous Work. Available online at: http://www.ilo.org/safework/areasofwork/hazardous-work/WCMS_110188/lang–en/index.htm

[B23] Iqbal K. Liu F. Gong C. X. Grundke-Iqbal I. (2010). Tau in Alzheimer disease and related tauopathies. Curr. Alzheimer. Res. 7, 656–664. 10.2174/156720510793611592 20678074 PMC3090074

[B24] Kilian J. Kitazawa M. (2018). The emerging risk of exposure to air pollution on cognitive decline and Alzheimer's disease - Evidence from epidemiological and animal studies. Biomed. J. 41, 141–162. 10.1016/j.bj.2018.06.001 30080655 PMC6138768

[B25] Langley M. Ghosh A. Charli A. Sarkar S. Ay M. Luo J.. (2017). Mito-apocynin prevents mitochondrial dysfunction, microglial activation, oxidative damage, and progressive neurodegeneration in mitopark transgenic mice. Antioxidants Redox Signaling 27, 1048–1066. 10.1089/ars.2016.6905 28375739 PMC5651937

[B26] Langley M. R. Ghaisas S. Ay M. Luo J. Palanisamy B. N. Jin H.. (2018). Manganese exposure exacerbates progressive motor deficits and neurodegeneration in the MitoPark mouse model of Parkinson's disease: relevance to gene and environment interactions in metal neurotoxicity. Neurotoxicology 64, 240–255. 10.1016/j.neuro.2017.06.002 28595911 PMC5736468

[B27] Leopold P. L. O'Mahony M. J. Lian X. J. Tilley A. E. Harvey B. G. Crystal R. G. (2009). Smoking is associated with shortened airway cilia. PLoS ONE 4:e8157. 10.1371/journal.pone.0008157 20016779 PMC2790614

[B28] Levesque S. Taetzsch T. Lull M. E. Kodavanti U. Stadler K. Wagner A.. (2011). Diesel exhaust activates and primes microglia: air pollution, neuroinflammation, and regulation of dopaminergic neurotoxicity. Environ. Health Perspect. 119, 1149–1455. 10.1289/ehp.1002986 21561831 PMC3237351

[B29] Liu B. Gao H. M. Hong J. S. (2003). Parkinson's disease and exposure to infectious agents and pesticides and the occurrence of brain injuries: role of neuroinflammation. Environ. Health Perspect. 111, 1065–1073. 10.1289/ehp.6361 12826478 PMC1241555

[B30] Lucchini R. G. Dorman D. C. Elder A. Veronesi B. (2012). Neurological impacts from inhalation of pollutants and the nose-brain connection. Neurotoxicology 33, 838–841. 10.1016/j.neuro.2011.12.001 22178536 PMC3387329

[B31] Maigler F. Ladel S. Flamm J. Ganger S. Kurpiers B. Kiderlen S.. (2021). Selective CNS targeting and distribution with a refined region-specific intranasal delivery technique via the olfactory mucosa. Pharmaceutics 13:1904. 10.3390/pharmaceutics13111904 34834319 PMC8620656

[B32] Massey N. Puttachary S. Bhat S. M. Kanthasamy A. G. Charavaryamath C. (2019). HMGB1-RAGE signaling plays a role in organic dust-induced microglial activation and neuroinflammation. Toxicol. Sci. 169, 579–592. 10.1093/toxsci/kfz071 30859215 PMC6542342

[B33] Massey N. Shrestha D. Bhat S. M. Kondru N. Charli A. Karriker L. A.. (2021a). Organic dust-induced mitochondrial dysfunction could be targeted via cGAS-STING or cytoplasmic NOX-2 inhibition using microglial cells and brain slice culture models. Cell Tissue Res. 384, 465–486. 10.1007/s00441-021-03422-x 33687557 PMC8154696

[B34] Massey N. Shrestha D. Bhat S. M. Kondru N. Charli A. Karriker L. A.. (2021b). Organic dust-induced mitochondrial dysfunction could be targeted via cGAS-STING or cytoplasmic NOX-2 inhibition using microglial cells and brain slice culture models. Cell Tissue Res. 10.1101/2020.07.01.182535 33687557 PMC8154696

[B35] McClendon C. J. Gerald C. L. Waterman J. T. (2015). Farm animal models of organic dust exposure and toxicity: insights and implications for respiratory health. Curr. Opinion Allergy Clin. Immunol. 15, 137–144. 10.1097/ACI.0000000000000143 25636160 PMC4783132

[B36] Molot J. Sears M. Marshall L. M. Bray R. I. (2021). Neurological susceptibility to environmental exposures: pathophysiological mechanisms in neurodegeneration and multiple chemical sensitivity. Rev. Environ. Health 000010151520210043. 10.1515/reveh-2021-0043 34529912

[B37] Nordgren T. M. Charavaryamath C. (2018). Agriculture occupational exposures and factors affecting health effects. Curr. Allergy Asthma Rep. 18:65. 10.1007/s11882-018-0820-8 30291457 PMC6644660

[B38] Nuber S. Petrasch-Parwez E. Winner B. Winkler J. von Hörsten S. Schmidt T.. (2008). Neurodegeneration and motor dysfunction in a conditional model of parkinson's disease. J. Neurosci. 28, 2471–2484. 10.1523/JNEUROSCI.3040-07.2008 18322092 PMC6671187

[B39] Oberdörster G. Elder A. Rinderknecht A. (2009). Nanoparticles and the brain: cause for concern? J. Nanosci. Nanotechnol. 9, 4996–5007. 10.1166/jnn.2009.gr02 19928180 PMC3804071

[B40] Ohmori H. Kanayama N. (2005). Immunogenicity of an inflammation-associated product, tyrosine nitrated self-proteins. Autoimmun. Rev. 4, 224–229. 10.1016/j.autrev.2004.11.011 15893716

[B41] Peters A. Veronesi B. Calderón-Garcidueñas L. Gehr P. Chen L. C. Geiser M.. (2006). Translocation and potential neurological effects of fine and ultrafine particles a critical update. Part Fibre Toxicol. 3, 13. 10.1186/1743-8977-3-13 16961926 PMC1570474

[B42] Poole J. A. Wyatt T. A. Oldenburg P. J. Elliott M. K. West W. W. Sisson J. H.. (2009). Intranasal organic dust exposure-induced airway adaptation response marked by persistent lung inflammation and pathology in mice. Am. J. Physiol. Lung Cell Mol. Physiol. 296, L1085–L1095. 10.1152/ajplung.90622.2008 19395665 PMC2692812

[B43] Prakash Y. S. Pabelick C. M. Sieck G. C. (2017). Mitochondrial dysfunction in airway disease. Chest. 152, 618–626. 10.1016/j.chest.2017.03.020 28336486 PMC5812762

[B44] Ransohoff R. M. (2016). How neuroinflammation contributes to neurodegeneration. Science 353, 777–783. 10.1126/science.aag2590 27540165

[B45] Saijo K. Crotti A. Glass C. K. (2013). Regulation of microglia activation and deactivation by nuclear receptors. Glia. 61, 104–111. 10.1002/glia.22423 22987512

[B46] Salvi A. Salim S. (2019). Neurobehavioral consequences of traffic-related air pollution. Front. Neurosci. 13, 1232. 10.3389/fnins.2019.01232 31824243 PMC6881276

[B47] Sarkar S. Malovic E. Harishchandra D. S. Ghaisas S. Panicker N. Charli A.. (2017). Mitochondrial impairment in microglia amplifies NLRP3 inflammasome proinflammatory signaling in cell culture and animal models of Parkinson's disease. npj Parkinson's Dis. 3, 30. 10.1038/s41531-017-0032-2 29057315 PMC5645400

[B48] Sarkar S. Nguyen H. M. Malovic E. Luo J. Langley M. Palanisamy B. N.. (2020). Kv1.3 modulates neuroinflammation and neurodegeneration in Parkinson's disease. J. Clin. Invest. 130, 4195–4212. 10.1172/JCI136174 32597830 PMC7410064

[B49] Seo Y. Kim H. S. Kang K. S. (2018). Microglial involvement in the development of olfactory dysfunction. J. Vet. Sci. 19, 319–330. 10.4142/jvs.2018.19.3.319 29032655 PMC5974513

[B50] Sethi R. S. Schneberger D. Charavaryamath C. Singh B. (2017). Pulmonary innate inflammatory responses to agricultural occupational contaminants. Cell Tissue Res. 367, 627–642. 10.1007/s00441-017-2573-4 28168324

[B51] Shin J. H. Kim I. D. Kim S. W. Lee H. K. Jin Y. Park J. H.. (2015). Ethyl pyruvate inhibits HMGB1 phosphorylation and release by chelating calcium. Mol. Med. 20, 649–657. 10.2119/molmed.2014.00039 25333921 PMC4365067

[B52] Shrestha D. Bhat S. M. Massey N. Santana Maldonado C. Rumbeiha W. K. Charavaryamath C. (2021). Pre-exposure to hydrogen sulfide modulates the innate inflammatory response to organic dust. Cell Tissue Res. 384, 129–148. 10.1007/s00441-020-03333-3 33409657 PMC8453448

[B53] Tani M. Glabinski A. R. Tuohy V. K. Stoler M. H. Estes M. L. Ransohoff R. M. (1996). *In situ* hybridization analysis of glial fibrillary acidic protein mRNA reveals evidence of biphasic astrocyte activation during acute experimental autoimmune encephalomyelitis. Am. J. Pathol. 148, 889–896. 8774143 PMC1861709

[B54] Trojsi F. Christidi F. Migliaccio R. Santamaría-García H. Santangelo G. (2018). Behavioural and cognitive changes in neurodegenerative diseases and brain injury. Behav. Neurol. 2018, 4935915. 10.1155/2018/4935915 30147810 PMC6083604

[B55] Ugrinova I. Pasheva E. (2017). HMGB1 protein: a therapeutic target inside and outside the cell. Adv. Protein Chem. Struct. Biol. 107, 37–76. 10.1016/bs.apcsb.2016.10.001 28215228

[B56] U.S. Geological Survey (2021). Agricultural Contaminants. Available online at: https://www.usgs.gov/mission-areas/water-resources/science/agricultural-contaminants?qt-science_center_objects=0#qt-science_center_objects: USGS. Available: https://www.usgs.gov/mission-areas/water-resources/science/agricultural-contaminants?qt-science_center_objects=0#qt-science_center_objects (accessed 22, October 2021).

[B57] Wright Willis A. Evanoff B. A. Lian M. Criswell S. R. Racette B. A. (2010). Geographic and ethnic variation in Parkinson disease: a population-based study of US Medicare beneficiaries. Neuroepidemiology 34, 143–151. 10.1159/000275491 20090375 PMC2865395

