# Supplementary material for: Mitoapocynin Attenuates Organic Dust Exposure-Induced Neuroinflammation and Sensory-Motor Deficits in a Mouse Model
Source: Front Cell Neurosci. 2022 Apr 13;16:817046. doi: 10.3389/fncel.2022.817046 (PMC9043522; doi:10.3389/fncel.2022.817046)
Supplement: Supplementary file 1 [file Data_Sheet_1.PDF]

Supplementary Material

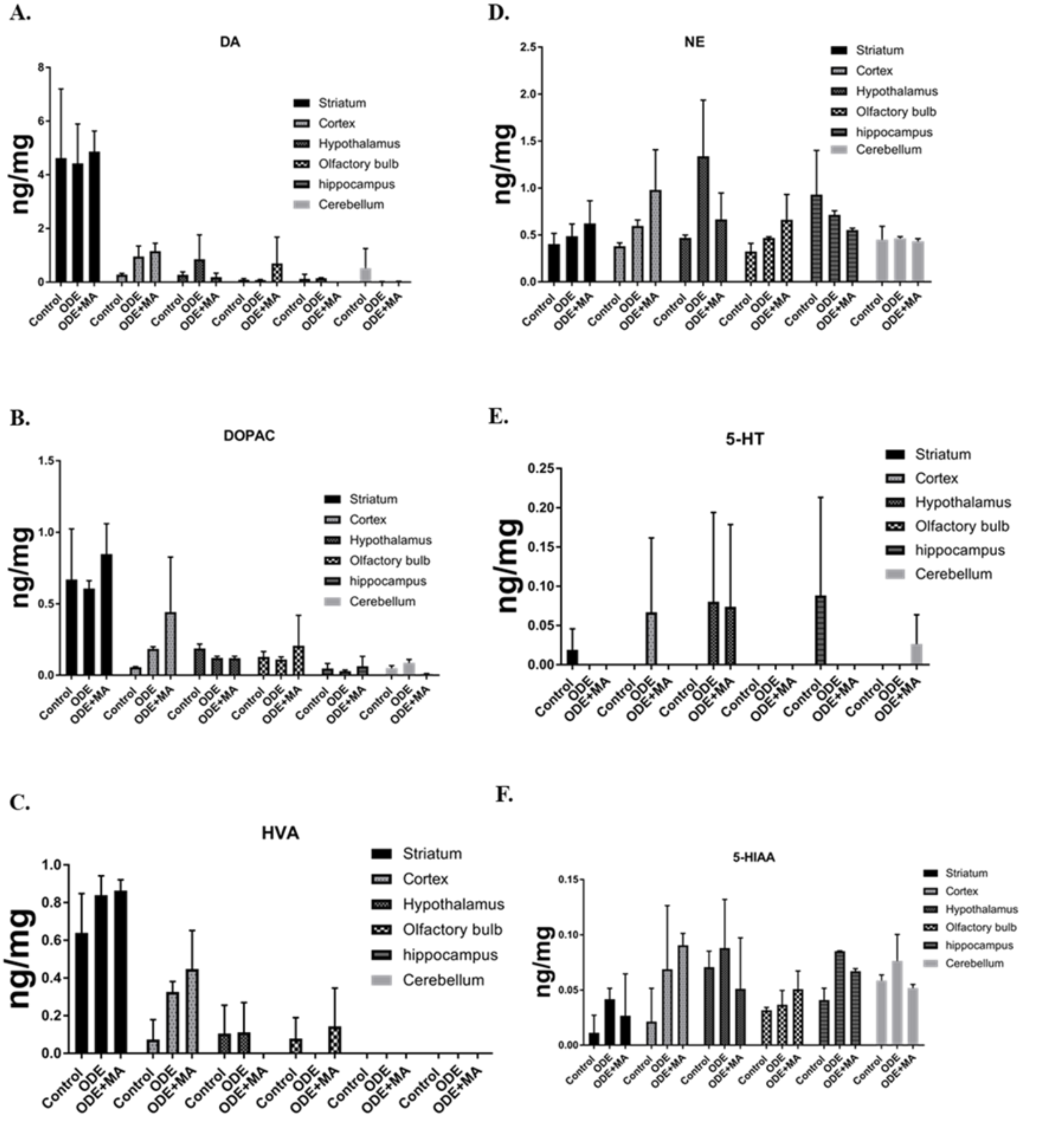

Supplementary Figure 1. Neurochemical Profile of control, ODE and ODE + MA exposed miceMice exposed to saline, ODE, and ODE with MA (3mg/kg) displayed moderate changes in the brain neurochemical profiles. Norepinephrine (NE) (D), Dopamine (DA) (A), Serotonin (5-HT) (E) and their respective metabolites, Dihydroxyphenylacetic acid (DOPAC) (B), Homovanillic Acid (HVA) (C) and 5-Hydroxyindoleacetic acid (5-HIAA) (F) were measured in various regions including Striatum, pre-frontal cortex, hypothalamus, olfactory bulb, hippocampus and cerebellum by HPLC-ECD.

Supplementary Table 1. Animal Treatments

Treatment group	Route	Days
Control (Normal Saline, 25 µL each nostril)	Intranasal	Monday to Friday
ODE (12.5 %, 25 µL each nostril)	Intranasal	Monday to Friday
ODE (12.5 %, 25 µL each nostril) + MA (3 mg/Kg) 50 µL total	Oral MA followed by Intranasal ODE	Monday/Wednesday /Friday
Fluorescent LPS (2 µg/ mice)	Intranasal	Monday to Friday

Supplementary Table 2. Antibodies used in Western blotting

Antibody	Catalogue	Dilution	Vendor
HMGB1	ab 79823	(1:15000)	Abcam
IBA1	ab 5076	(1:500)	Abcam
Phospho-Tau (Ser202, Thr205)	MN1020	(1:1000)	Thermo Fisher Scientific

Supplementary Table 3. Antibodies used for ICC

Primary Target (Host species)	Catalog #	Secondary Antibody	Catalog #
HMGB1 (Rabbit)	ab 79823	Anti-Rabbit (Cy3 conjugated) raised in donkey	711-165-152
3-NT (Mouse)	ab 61392	Anti-Mouse (FITC conjugated) raised donkey	715-095-150

Iba1 (Goat)	ab 5076	Anti-Goat (Cy3 conjugated) raised donkey	711-165-147
GFAP (Mouse)	G3893	Anti-Mouse (FITC conjugated) raised donkey	715-095-150
Phospho-Tau (Ser202, Thr205) (Mouse)	MN1020	Anti-Mouse (FITC conjugated) raised donkey	715-095-150
NueN (Rabbit)	ABN78	Anti-Rabbit (Cy3 conjugated) raised in donkey	711-165-150
Acetylated α tubulin(Mouse)	Ab24610	Anti-Mouse (Cy3 conjugated) raised in donkey	711-165-150

Supplementary Table 4 Symbols (Asterisk or Hashtag) and Corresponding p-Values

Symbol	p-value
* or #	≤.05
** or ##	≤.01
*** or ###	≤.001
**** or ####	≤.0001
